# Exploring the complexities of slum vulnerability in Haryana, India: a qualitative research journey into economic, social, physical, and health dimensions

**DOI:** 10.1080/17482631.2024.2432692

**Published:** 2024-12-15

**Authors:** Manmeet Kaur, Abhishek Sharma, PP Vijin, Rupinder Kaur, Rajbir Kaur, G. Anupama, Prabhjot Singh, V.K Bansal, Nidhi Sharma, Mona Sahni, Jyoti Gupta, Pvm Lakshmi, Rajesh Kumar

**Affiliations:** a Health Equity Action Learnings, Chandigarh, India; b Postgraduate Institute of Medical Education and Research, Chandigarh, India; c Health Department, Government of India, Haryana, India; d National health Mission, Government of India, Haryana, India; e National Urban health Mission, Government of India, Haryana, India

**Keywords:** Vulnerabilities, Slums, qualitative research, focused group, health outcomes

## Abstract

**Purpose:**

The study explores the conditions contributing to slum dweller’s vulnerability to poor health and examines interplay between economic, physical/infrastructural, and social factors affecting health status to inform policy and programme.

**Methods:**

The methodology deployed for data analysis was mixed deductive-inductive. A deductive framework was adapted for categorizing the data into four broad themes: Economic, Physical/Infrastructure, Social, and Health. Using Braun and Clarke’s principle, we also mapped sub themes based on researchers’ insights with the experiences shared by the slum populations. The study was conducted across 13 districts in slum areas to gather information from vulnerable groups based on the 40 focus group discussions.

**Findings:**

Poverty serves as a primary driver of domestic/internal migration from rural to urban areas, aggravating issues such as illiteracy, unemployment, and inadequate living conditions, which predispose slum dwellers to various health problems. Limited access to food, poor water quality, and improper waste disposal further compound health risks. People living in slums face economic, social, and physical vulnerabilities leading to health vulnerability and outcome.

**Conclusions:**

Enhancing the health and well-being of slum dwellers requires adopting an integrated and comprehensive approach of policy intervention, community mobilization, and multisectoral intervention.

## Introduction

Movements of the human population from rural to urban areas is one of the rapid forms of demographic transition (United Nations, 2018). Currently, over half of the global population resides in cities, and it is projected that this proportion will rise to 68% by 2050 (Government of India, 2011). In India, migration is often driven by the pursuit of education, healthcare, and access to basic infrastructure facilities. Others migrate in search of work as there is land scarcity, labour demands, poor occupational conditions, and poverty. Housing in urban areas especially in countries like India is unaffordable for internal migrants from rural areas in search of job often end up living in slums (Migration, 0000).

The United Nations Program on Human Settlements (UN-HABITAT) defines slum as “a contiguous settlement where the inhabitants are characterized as having inadequate housing and basic services. A slum is often not recognized and addressed by the public authorities as an integral or equal part of the city” (Urban Secretariat & Branch, 2002). These settlements are typically located on government or private land, and often lack basic infrastructure and services such as housing, sanitation, clean water, and electricity. Such living conditions expose slum dwellers to numerous vulnerabilities, as many are employed in precarious occupations such as daily wage labour, street vending, rickshaw pulling, construction work, and other informal sectors (Braun, V., & Clarke, V. 2006; Titiunik, R. 2009).

According to the 2011 census, India had approximately 65 million residents living in slums; around 17% of the urban population Slum dwellers frequently experience prejudice and marginalization, which limits their educational and employment opportunities, and access to healthcare (Government of India, 2011; United Nations, 2012). All these barriers act together to make people vulnerable to poor health and health outcomes.

Vulnerable groups are disadvantaged because of which they fail to make and implement informed decisions about their life (Shivayogi, 2013). The poor, ethnic minorities, low-income children, the elderly, the homeless, and those with chronic health conditions are recognized as the vulnerable groups (Mberu et al., 2016). Despite better healthcare accessibility, both morbidity and mortality rates are higher in slum areas compared to rural or urban areas (Mberu et al., 2016).

The grand social and economic theories such as structural, functional and conflict and meso e.g., demographic transition or micro push and pull factors have explained why people migrate and what kind of socio-psychological and economic adversities can be expected (A portrait of the chronically ill in America 2001; De Haas, 2021). Researchers have tested these theories empirically using mostly positivist approach and provided evidence supporting several aspects of these theories, as well as identifying limitations and gaps that warrant further investigation (Park et al., 2020). Following the critical realist theory that challenges the empiricism and positivism and helps in uncovering the underlying structures, the present study examines the vulnerabilities of people living in slums.

National Urban Health Mission (NUHM) of Haryana state in collaboration with PGIMER (Post Graduate Institute of Medical Education and Research), Chandigarh explored the conditions that make slum dwellers vulnerable to poor health. This research contributes to existing literature by providing a comprehensive analysis of the factors contributing to the vulnerability of slum dwellers to poor health. The present study also examined the healthcare service availability, utilization, and health issues faced by the slum population to answer the following research questions:

- How do economic, physical/infrastructural, and social vulnerabilities affect health status?
- What kinds of healthcare services are accessible and offered to slum populations?
- What barriers does the slum population face in accessing healthcare services face?

To answer these questions, various models of vulnerability and health outcomes were reviewed and a general model of vulnerabilities (Braun & Clarke, 2006) and health outcomes has been applied to develop a contextual model to be followed for action.

## Methods

The present study is a qualitative component of the larger mixed-methods research involving concurrent use of both qualitative and quantitative data. It focuses on the qualitative findings, while the quantitative outcomes will be disseminated separately in another publication. This qualitative study explores the living and working conditions, health issues, and healthcare services availability to the people living in slums.

### Study Setting

The present research was conducted in selected cities across 13 Districts within the state of Haryana, India. Districts serve as administrative divisions within a state. Notified slums in each District were identified based on available official data. The list of selected groups for FGD’s was provided by NUHM, Haryana which included specific vulnerable groups such as ragpickers, elderly, commercial sex workers, domestic workers, minority population, daily wager, and factory workers (and others as specific to the city). For data collection, at least two locations within each city were selected from among the notified (officially declared) slums.

### Sampling

A purposive sampling was applied to collect the data among the community members such as rag pickers, unorganized labourers, female sex workers, truck drivers, domestic workers (household help), leprosy patients, nomads, and refugees.

Local healthcare staff, including ANMs (Auxiliary Nurse and Midwife) and ASHAs (Accredited Social Health Activists), assisted in identifying geographic locations and approaching individual participants for recruitment in the study. Participants were given the opportunity to ask questions and clear any doubt before agreeing to participate for the research, thus ensuring that they were well informed about the objectives and procedures of the study.

The group size of Focus Group Discussions (FGDs) ranged between 8–15 participants. The FGD participants included both males and females; generally, a single FGD however comprised members of only one gender. To cover, both genders, all age groups, and occupational categories (rag pickers, unorganized labourers, female sex workers, truck drivers, domestic workers (household help) a total of 53 FGDs were conducted. Four out of 53 FGD’s were excluded from the study due to poor audio quality and, nine were excluded because of data saturation, and finally, 40 FGDs were included in the analyses. A total of 30 FGDs were proposed to encompass all localities with diverse housing (3), occupational groups (8), genders (2), ages (3), and specific ethnic groups. The goal was to conduct a minimum of two FGD’s per category, but more were ultimately conducted to ensure saturation.

### Data Collection

The data was collected by the staff of National Health Mission (NHM), Haryana after training them on a pretested topic guide provided by the PGIMER staff. The topic guide covered issues such as housing conditions, working conditions, availability of clean drinking water, toilet and waste disposal management, healthcare accessibility, health issues, sanitation and hygiene services.

The data was collected after receiving ethical clearance from the Institute’s ethical committee (PGI/IEC/2018/001019 dated 10/08/2018). FGDs were audio-recorded after obtaining both written and verbal consent from the participants. Notes were taken by the note taker who accompanied the moderator. The moderator acknowledged that the Information will be kept confidential and agreed not to use or disclose identity of the participant in the research. By keeping the data confidential and anonymizing identities of the participants, the researchers took appropriate steps to safeguard the information from disclosure or unauthorized use.

### Data Analysis

Audio-recorded data were transcribed into a textual document, and the transcriptions were reviewed for accuracy. The data recording and notes were read twice to familiarize with the information. A senior researcher led the analysis, with assistance from another researcher in familiarizing themselves with the data. The methodology deployed for data analysis was mixed deductive-inductive that balanced the theory-driven categorization of data with the adoption of data-driven insights (Braun & Clarke, 2006; Fereday & Muir-Cochrane, 2006). A deductive framework was adapted for categorizing the data into four broad themes: Economic, Physical/Infrastructure, Social, and Health. Such a categorization emerged from available literature related to the vulnerabilities of marginal urban populations that offers clarity to the overall analysis (Cattaneo et al., 2009; Farmer, 2004). Each of these broad themes were chosen to capture the dimensions of vulnerability based on the given context of slum dwellers. These deductive themes provided a structure; however, inductively drawn sub themes were context specific and emerged from participants’ shared experiences. Inductive coding enabled probing deeper into sub-themes and finer nuances that were not initially anticipated and proved crucial to capture unique vulnerabilities of the slum dwellers in India. Data analysis were done through NVivo 12.

## Findings

The majority of the respondents had migrated from other states such as Uttar Pradesh, Bihar, Rajasthan, and Madhya Pradesh that are agricultural and relatively poorer to other states of India or other districts of Haryana in search of livelihood. The details related to various vulnerabilities experienced by the slum dwellers are categorized in Table I. Themes and sub-themes provide detailed descriptions of different vulnerabilities leading to health outcomes. Following the clues from literature, we decided to start with the economic vulnerability.

**Table I.** Vulnerabilities affecting health of slum dwellers in Haryana, India.

Vulnerabilities	THEMES/SUB THEMES	DESCRIPTION	QUOTES	
ECONOMIC	Precarious Employment Conditions	Occupations such as rag picking, unskilled labour, truck drivers or seasonal vendors are prevelant.	“*We are rag-pickers, we collect garbage and provide food to our children” (FGD33M)*	
	Insufficient Daily Wages	Income ranges between INR 100 to 300 per day. These are below the minimum daily wage rates fixed by the Government for specific districts	“*We hardly earn 200–300 rs per day which is not enough to feed food to family” (FGD8M)*	
PHYSICAL/INFRASTR-UCTURAL SOCIAL	Substandard Housing and Proximity to Environmental Hazards	Majority of slum dwellers stay in Kacha or temporary structures with tin roofs, mud brick walls and mud floor	*I have a kacha house and sometimes during rainy season there is seepage from the ceiling” (FGD15F)*.	
	Inadequate Water, Sanitation, and Waste Management	Dwellers complained about the poor quality of water supplied. Common toilets for such a large number of people poses health risks such as spread of infectious diseases. In absence of the garbage disposal system, slum dwellers are forced to dispose garbage in open places	*“Tap water is very dirty and unfit for drinking but we have no other option,” (FGD2F)*	
	Pervasive illiteracy	More than 80% of the respondents were literate	“We are mostly illiterates” (FGD35M)	
	Irregularity in Public distribution scheme	Despite being enrolled under Public distribution system (PDSS), only few families are able to avail all the benefits of this scheme.	*“We only get wheat; our card is green which means we are not eligible for all items” (FGD28F)*	
	Limited Awareness and Access to Health Insurance	The awareness and access related to Ayushman Bharat scheme (Public Health Insurance scheme) is poor among the dwellers.	*“we don’t know how to get health insurance, we are barely hand to mouth, so how can we have insurance” (FGD21M)*	
HEALTH	Availability and barriers in healthcare utilization	Some of the slum dwellers preferred private healthcare due to two main reasons; one, the long waiting hours in government hospitals, two, non-availability of transport to reach to the health facility	*“Government hospitals areoverburdened” (FGD23F)*	
	Prevalence of Communicable Diseases and Environmental Health Risks	Diarrhea, viral fever, dengue, malaria, seasonal flu, skin infection, typhoid, and tuberculosis were the most common diseases mentioned in most FGDs	*“No garbage disposal mechanism. Community is at high risk of diseases and need immediate intervention” (FGD30F)*	

Note: Identifiers for Quotes: FGD number and gender (*M*- Male, F- Female).

## Economic vulnerability

Most of the domestic migrants living in slums started their journey to search for jobs due to inadequate economic circumstances in their rural homelands, which posed challenges in supporting their families. Their employment conditions in the urban areas, as gathered from FGDs, are presented below. The economic fragility of these migrants was a profound issue that pertained to very low, irregular incomes, unavailability of basic services, and insecure living conditions. The informal sector occupied a large share of employment for slum dwellers, which was poorly paid, had less stability, and provided no social security or benefits.

### Precarious Employment Conditions

Most of the slum dwellers were daily wagers, truck drivers, seasonal vendors, and rag pickers. These jobs were irregular; most of the time, they were engaged for only half of the month (15 days). Truck drivers reported that they operated rented vehicles and were often out of employment for more than ten days in a month. The majority of the respondents had migrated from other states (such as Uttar Pradesh, Bihar, Rajasthan, and Madhya Pradesh) or other districts of Haryana in search of livelihood. Rag pickers, seasonal vendors, and daily wagers suffered day in and day out from the gruelling hardships of unpredictable working hours in precarious, physically exhausting environments without any form of guaranteed income. They lived every day in fear of not making enough for themselves to provide for their basic needs the next day, constantly facing the widespread fear of hunger and scarcity while being held in a perpetual grip of uncertainty that consistently denied them a stable financial arrangement. Consequently, due to this irregularity, they did not send their children to schools and engaged them in wage work. Most children were withdrawn from school to help their families undertake daily occupations, especially rag picking jobs, where children accompanied their parents during the day. Very often, women and children were involved in similar jobs as their male counterparts.

Yes, these children sitting here are serving as domestic helps, rag pickers and sometimes vendors, they have a compulsion to work as no one here has any kind of permanent employment, rag picking is the only occupation (FG31F)

### Insufficient Daily Wages

Study participants earned INR 100 to 300 per day, which was insufficient to provide adequate food and other amenities for their families, resulting in substandard living conditions and an unhealthy environment. Due to their lack of competence and inability to find regular work, the majority of them found themselves in worse financial situations. In many households, women contributed equally to the economic pool. With both partners working, a household could earn more than INR 300 per day. Rag pickers and daily wagers reported difficulties in meeting their daily food requirements and had days when they could only afford one meal.

Our father migrated to Karnal from UP(other state), I was born and raised here, we are not able to earn enough and are not able to provide better food or housing to our children (FGD5M)

This inability to afford simple basics like nutritious food and clean water also forced them to opt for a choice between short-term survival and long-term well-being. The lack of financial security prevented them from planning or elevating their living standard, as they were involved in a cycle of hand-to-mouth operations, where every day is a struggle to make ends meet and left no chance for uplift and possibility of a better tomorrow. Low-income pushed slum population into the web of poverty and impacted their health because of a different level of hardship and limited access to food, and a healthy environment.

## Physical/infrastructural vulnerability

Slum dwellers often had temporary structures for dwellings that were poorly built, lacking space and ventilation. Poor water and sanitation services put the population at risk of various diseases and affected the overall development of children.

### Substandard Housing and Proximity to Environmental Hazards

Dwelling or housing can have substantial impacts on health in terms of structure, exposed to environmental hazards. More than half of the participants from 13 districts resided in temporary structures known as kutcha houses or shanties. These often had mud walls with tin roofs, while only a few had brick houses with cement and concrete roofs. Most of the slum dwellers lived in overcrowded areas characterized by small lanes with unhygienic conditions, lacking proper ventilation and drainage systems. Rag pickers and daily wagers were housed in shanties, while truck drivers, nomads, domestic labourers, and leprosy patients lived in rented concrete houses. Shanty dwellers had no electricity or water connections. Heavy rains and windy weather posed dangers to their lives as their temporary structures often failed to withstand these conditions. Participants also mentioned that concrete houses were devoid of proper ventilation and space, causing health issues such as skin diseases and asthma.

Most of the slums were located near open drains or polluted water bodies, such as local rivers or ponds, making them vulnerable to floods. Open drains and waterlogging in the nearby surroundings resulted in the breeding of mosquitoes, rendering them susceptible to various diseases. Slum dwellers felt unsafe during the rainy season due to the proximity to open drainages that frequently flooded, high-tension wires, and nearby railway lines. The combination of faulty housing, overcrowding, and lack of sanitation increased their vulnerability to disease.

We stay near the nallah which has all the garbage of the city and gets flooded every time when raining heavily, the flooded water gets mixed with garbage and sewage water, we have to clear it ourselves, no one is there to help (FGD28F)

### Inadequate Water, Sanitation, and Waste Management

Access to WASH (Water, sanitation, and hygiene) played a vital role in maintaining good health. Slum dwellers revealed that the water distributed was of poor quality and could not be consumed. Rag pickers and daily wagers lacked a safe source of drinking water and were forced to use water from nearby households or community taps or purchase bottled water from local shopkeepers. Despite their low income, slum dwellers spent their hard-earned money on pure drinking water. The majority of respondents complained that the water supply had a typical foul smell resembling sewer water, making it challenging to drink or use for cooking purposes. In every district where the government supplied water, reports indicated that common taps served over 20 households. Slum dwellers had long complained about the water problem, but little was done to address it.

Sewer water is mixed in drinking water, it is very unfit to drink, we complained about it but no action was taken by any one (FGD35M)

Common toilet facilities were available in almost all the slum locations, except for shanties in 4 out of 22 districts, where inhabitants resorted to open defaecation. Most families had a common toilet, while very few had personal toilets. The majority of slum locations marked the absence of public toilets. Reports indicated that public toilets were often poorly maintained, discouraging slum dwellers from using them. Irregular supply of water was one of the main reasons for the poor maintenance of public toilets. Some locations had one toilet for more than 15 families.

Public toilets are not clean; we cannot use them (FGD9M)

Most of the slum dwellers reported that there was no proper mechanism for garbage disposal, and they had to manage waste disposal on their own. In the absence of garbage disposal systems, they were forced to dispose of garbage in open locations such as unoccupied plots, drainage pipes, and pavements. Collection of garbage was irregular which often left them with only one option i.e., open disposal of garbage.

No one comes to take garbage, we have no other option but to dispose it in open grounds or drainage pipes, MC has not even provided the dustbins in the locality, I personally dont like to litter but what can we do, we have complained many times to the authorities but no one listens” (FGD23F)

## Social vulnerability

Among slum populations, widespread illiteracy remained a serious problem, limiting prospects for social and economic growth and perpetuating the cycle of poverty. Lack of education prevented most illiterate people from securing permanent jobs with decent wages, leaving them to work for minimal pay. Such individuals were barely able to comprehend information regarding social welfare entitlements, healthcare, and upward mobility opportunities.

The government provided social welfare schemes such as the Public Distribution System (PDS) for food and health insurance. PDS focused on the distribution of food grains in scarcity areas (18). The Government of India started a social health insurance scheme named Ayushman Bharat Pradhan Mantri Jan Arogya Yojana, which aimed to provide free health insurance coverage for low-income earners in the country (19). Access to PDS played a critical role in ensuring food security for slum households, which often struggled with inadequate income to meet basic dietary needs. Meanwhile, access to health insurance through Ayushman Bharat offered these households some measure of financial relief during health emergencies, which were otherwise a major economic burden.

### Pervasive illiteracy

More than 80% of the respondents in all 13 districts were illiterate, and the rest had only completed elementary education, rendering them functionally illiterate. The illiteracy and low education levels pushed them into unskilled labour within the unorganized sector. Due to illiteracy, they often resorted to temporary employment to make ends meet, leaving no time to prioritize their children’s education, resulting in a high rate of school dropouts among their children.

They mentioned that their children dropped out of school to contribute economically to their families by engaging in daily wage labour. For instance, children of rag pickers often accompanied their parents during the daytime, providing an extra hand in the job. Despite free schooling in government schools, the dropout rates among children of rag pickers remained high, followed by children of daily wagers.

### Irregularity in Public distribution scheme

The Public Distribution System (PDS) to ensure food for families below the poverty line was functional in all the districts, and the majority of participants were enrolled in the PDS. However, due to eligibility criteria, only a few families could avail themselves of all the benefits of the scheme. Rag pickers and daily wagers who had migrated from other states were disqualified from receiving full benefits under the PDS, making it difficult for them to secure food through the system.

Families availing of PDS services complained about the poor quality of rations provided. Given the financial conditions of slum dwellers, they required regular services under PDS, yet various rules and regulations prevented them from accessing all benefits.

We only get wheat and that too of vary bad quality (51F)

Many families relying on the PDS and other government schemes complained of receiving poor-quality food. Though the PDS was designed to end hunger and ensure food security for many communities, it often provided substandard provisions that barely met nutritional needs. These shortfalls were especially concerning as they affected the health of families, particularly children, who required balanced nutrition for proper growth and development. The issue of food quality was not just a source of discontent; it reflected deeper flaws in the PDS system. Most families felt ignored and unheard, as their complaints were frequently dismissed and left unaddressed.

### Limited Awareness and Access to Health Insurance

Very few participants were enrolled in the Ayushman Bharat scheme, a government-aided health insurance initiative, with many lacking awareness of the procedures for accessing its benefits. Most slum dwellers were unaware of the Ayushman Bharat scheme or any other health insurance options. Some slum residents mentioned that they were unable to enrol in health insurance schemes due to the non-availability of a PDS card. The majority of community members were not enrolled in any health insurance scheme, and awareness and access related to the Ayushman Bharat scheme were poor among participants. Only a few participants reported having health cards for Ayushman Bharat, but they were unfamiliar with the process for availing themselves of the scheme’s benefits. Despite the Ayushman Yojana being launched with considerable enthusiasm, it failed to reach the poor who actually qualified for this health insurance. Rag pickers, unskilled workers, and jhuggi dwellers were not enrolled in any form of health insurance.

Ayushman Bharat facility is given to those who have the Ration card. As we do not have Ration card so we do not have the facility (FGD11F)

## Health vulnerability

### Health vulnerability

The above-mentioned vulnerabilities–economic, physical/infrastructural, and social–illustrated the multifaceted challenges faced by slum dwellers, particularly concerning their health. Economic vulnerability, stemming from low education levels and irregular employment conditions, hindered access to necessities such as food and housing. Physical and infrastructural vulnerabilities, characterized by inadequate housing and poor access to water and sanitation services, exposed slum dwellers to various health hazards. Social vulnerability exacerbated these challenges, as limited enrolment in social welfare schemes and dissatisfaction with available services highlighted the barriers to accessing essential support systems.

### Availability and barriers in healthcare utilization

Access to healthcare services affected the health outcomes of the population. In the majority of slum locations, public healthcare facilities were located within a range of five kilometres. However, barriers to utilizing public healthcare facilities led some residents to prefer private healthcare for two main reasons: long waiting periods in government hospitals and the lack of transport facilities. Slum dwellers from District Sirsa, Panchkula, and Gurgaon reported that government dispensaries were located far from their homes, making them inaccessible, especially during night-time.

Common health problems reported by community members included diarrhoea, viral fever, dengue, malaria, seasonal flu, skin infections, typhoid, and TB. The participants identified poor quality drinking water and unhygienic surroundings as the root causes of these health issues. The majority of respondents lived hand to mouth, so they could not afford to waste a day’s wage waiting in government hospitals for their turn and preferred to consult quacks or private care facilities. The active role of NGOs was absent in all districts except for District Panchkula, District Gurgaon, Ambala, and Karnal, where some NGOs were operational. Despite the need for assistance from both government and non-government sectors, it was surprising that limited numbers of NGOs worked in slum locations.

We cannot waste a day’s wage waiting in the government hospital so prefer to consult quacks or private care facilities (FGD3M)

### Prevalence of Communicable Diseases and Environmental Health Risks

Diarrhoea, viral fever, dengue, malaria, seasonal flu, skin infections, typhoid, and tuberculosis emerged as the most common diseases mentioned in most FGDs. Participants identified poor quality drinking water and unhygienic surroundings as the root causes of these health problems. None of the participants mentioned non-communicable diseases such as diabetes or hypertension.

Due to overflow of drainage water, our community members suffer from malaria, dengue and other diseases (FGD24M)

## Discussion

The purpose of this study was to explore and explain the susceptibilities that people living in urban slums face and how these affect the health outcomes. The findings of the study have established the conditions in which urban poor live and work, and how it affects their health. We carried out a concurrent mixed method study to understand the vulnerabilities of people living in the slums of the state of Haryana. Only the qualitative findings have been used to explore and explain the social, economic and physical, health vulnerabilities leading to poor health outcomes.

The findings of the study suggest that there is a vast gap in the economic, living and health conditions of slum dwellers as compared to other urban residents. These conditions do not allow poor people to break the cycle of poverty for which they had migrated from the rural areas to urban.

Literature review established that agriculture and Industrial policies lead to poverty, poor literacy and education, and migration to urban areas (Causes of Urban Poverty in India). Poor skills lead to daily wage work in the unorganized sector and poor housing giving way to health vulnerability and poor health outcomes (Jayanthakumaran et al., 2019; Parida, 2019). This includes economic vulnerabilities e.g., working in the unorganized sector without social security, physical vulnerabilities, such as exposure to natural hazards, and social vulnerabilities, such as availing benefits of social welfare schemes leading to health vulnerability i.e., the risk of experiencing negative health. These vulnerabilities stem from various factors such as poverty, inadequate healthcare access, poor living conditions, limited education, social exclusion, environmental hazards, and lack of support networks. All these together lead to health vulnerability and poor health outcomes.

The migrants often face economic vulnerabilities when they join the urban job market with lower education and skill levels (Corburn et al., 2020; Das et al., 2021; Mberu et al., 2014). This often leads to them working in the unorganized sector, which typically offers lower income levels and limited job security. Additionally, factors such as inadequate housing and poor water, sanitation, and hygiene (WASH) infrastructure further exacerbate health and well-being challenges among migrants and other urban poor populations (Water supply, sanitation, and hygiene).

To address some of these problems, the government has implemented social welfare schemes such as the Public Distribution System (PDS) for food security (Public Distribution Scheme) and the Ayushman Bharat Pradhan Mantri Jan Arogya Yojana to provide free health insurance coverage for low-income earners (Ayushman Bharat - Health and Wellness Centre). However, despite the existence of these schemes, many slum dwellers and urban poor individuals are unable to benefit from them due to difficulties in proving their identity and poverty status through official documents.

This situation underscores the importance of not only implementing social welfare programmes but also ensuring that they are accessible and inclusive. Simplifying documentation requirements, increasing awareness about available schemes, and providing support to help individuals navigate bureaucratic processes can help ensure that vulnerable populations, including migrants and slum dwellers, are able to access the support they need to improve their economic stability, health outcomes, and overall well-being.

The National Rural Health Mission launched by Government of India in 2005 aimed to alleviate the health problems faced by the rural Indian population (National Rural Health Mission). Thereafter, in 2013, National Urban Health Mission was unveiled for providing equal opportunities to socio-economically weaker including urban slum populations (National Urban Health Mission). The mission that was started about 10 years ago does not seem to be reaching out to slums. Our study explored that urban slum caters to leprosy patients, dependent on the charity and grants provided by NGOs (Non-Governmental Organizations) for their livelihood. They also experience social stigma which serves as a significant barrier to getting employed and seeking health services. Therefore, they remain unemployed. Most of the unemployed youth in slums were because of one or the other kind of disease or disability. Their health condition was acting as social and economic vulnerability.

### Drivers of rural urban migration and slum growth

Rural-to-urban migration, driven by economic, social, and environmental factors, often leads to slum growth due to inadequate urban planning and affordable housing. Consequently, slum dwellers face significant health risks, including poor sanitation, contaminated water, and substandard housing conditions, exacerbating their vulnerability to communicable and non-communicable diseases (Edition, 2011). Most of the slum dwellers had the same explanation of their roots and reasons of migration that they did not miss in their introductory remarks.

The migration of populations from rural to urban areas, coupled with the inability of city administrations to plan and provide affordable shelter or housing for low-income groups, has resulted in massive slum growth (Ooi & Phua, 2007). About two-fifths of cities/towns in India have reported slums, including those in the state of Haryana.

### Challenges faced by slum dwellers and policy failures

The qualitative findings of study exposes that the slum population consists predominantly of individuals who have migrated from rural areas seeking to alleviate their economic challenges but they have become entrenched in social and physical conditions within the slums that exacerbate their vulnerability to health issues. Slum dwellers have always been and continue to be more vulnerable to poor health outcomes as are clear from the findings of our study. Their susceptibility to negative health impacts arises from a combination of factors, including poverty, inadequate healthcare access, poor living conditions, limited education, social exclusion, environmental hazards, and lack of social support networks.

The majority of slum dwellers in our study are living in unhealthy living conditions marked by faulty drainage systems and openly disposed garbage. Government water supply schemes are functional in some areas but the quality of water received is very poor and causes different health problems. The poor living conditions such as shanties, poor drainage and wastage disposal make them vulnerable to communicable diseases and the poor availability of nutritious food and stress of insecure job lead to non-communicable diseases. Health issues like diarrhoea, viral infections, and more, as well as dengue fever, malaria, and skin infections are common due to these conditions.

People who migrate to urban areas are reported to be poorest based on their inability to spend (PWC India, 2015). Not only the bad housing, the slum dwellers have been the victims of unjustifiable access to the health and other social well-being services because of the poor implementation of policies (Banerjee et al., 2012). This has resulted in a poor health status of urban slum population in India especially among children marked by higher rates of undernutrition, neonatal and under- five mortality and low rates of coverage under universal immunization (Usmani & Ahmad, 2018). The determinants of health among the urban slum population are economic conditions, social conditions, living environment, access to public healthcare, mobility, morbidity, and literacy (Banerjee et al., 2012; Pawar et al., 2008).

The present study advocates for effectively responding to the needs of the urban poor for which big economic and social policy shifts are needed. All types of vulnerabilities reflected in this study calls for use of community participatory along with multisectoral approach for developing plans so as to respond to the needs of people and reduce health vulnerability and wellbeing of all.

The National Food Security Act, The Pradhan Mantri Krishi Sinchai Yojana (PMKSY), The National Mission Sustainable Agriculture (NMSA) and Mahatma Gandhi National Rural Employment Guarantee Act 2005 (MGNREGA) etc. implemented over the years to support and boost the agriculture sector have not been able to help the poor slum population. The farmers in India with small landholdings find it difficult to survive and are moving to the urban areas in search of jobs. Industrialization and urbanization did attract the rural population, but the development policies could not offer social security and failed to deliver on fundamental human rights such as housing, education, and health (Agriculture - National Mission for Sustainable Agriculture).

### Policy interventions and vulnerability framework

The qualitative analyses in the study provides causes and contributors to vulnerabilities. It explains that how these vulnerabilities lead to poor health outcomes. The participants in this study could not explain communicable diseases while there is enough evidence of rising hypertension and diabetes among poor. The findings suggest that the policies need a revision from time to time to address the emerging issues of the slum population. This can be achieved through regular monitoring and evaluation of interventions and their effectiveness to redress the vulnerabilities and saving populations from the emerging ones.

## Conclusion

This study highlighted major vulnerabilities which the urban slum population is exposed to in Haryana, specifically in terms of social, economic, physical, and health issues. Though several government schemes and programmes aid in lessening these problems, issues regarding accessibility and documentation are some of the handicaps that affect its effectiveness in general. However, it is only by adopting an integrated and comprehensive approach of policy intervention, community mobilization, and multisectoral intervention in such areas that health and well-being among slum dwellers will be enhanced. Inclusive programmes are needed to improving housing, water, sanitation, and societal conditions for improved health outcomes.

## Strengths and limitations

This is a comprehensive study based on large dataset covering 13 Districts, 3,07,259 people for the quantitative component and the qualitative findings are based on 40 FGD’s with 454 participants. Though extensive literature review was carried out but the national and state level policy and programme review is limited to this study. Policy analyses and a programme review of different sectors such as agriculture, industry, urban and rural development, education is required before launching multisectoral action.

## Supplementary Material

FGD Tool.pdf

## Data Availability

The authors attest that the interview transcripts contain the information needed to support the findings of the study. The authors confirm that, for ethical and security reasons, they are unable to make interview transcripts and internal administrative documents publicly available. The participants of this study did not consent to have their transcripts made publicly available, as they contain personal information which could be used to identify the participants. Requests for these data can be sent to the corresponding author [abhishekanthropu@gmail.com]. Access to these restricted data will be granted where de-identification can be adequately achieved to protect the privacy and confidentiality of the respondents and any mentioned individuals and institutions, and where the proposed use is seen as relevant to the nature of the data.

## References

[cit0001] Agriculture. Retrieved March 19, from https://www.india.gov.in/topics/agriculture

[cit0002] Ayushman bharat - health and wellness centre. Assessed on January 18 from http://ab-hwc.nhp.gov.in

[cit0003] Banerjee, A., Bhawalkar, J. S., Jadhav, S. L., Rathod, H., & Khedkar, D. T. (2012, January). Access to health services among slum dwellers in an industrial township and surrounding rural areas: A rapid epidemiological assessment. *Journal of Family Medicine and Primary Care*, 1(1), 20. 10.4103/2249-4863.94444 PMC3893946 24478995

[cit0004] Braun, V., & Clarke, V. (2006). Using thematic analysis in psychology. *Qualitative Research in Psychology*, 3(2), 77–11. 10.1191/1478088706qp063oa

[cit0005] Cattaneo, M. D., Galiani, S., Gertler, P. J., Martinez, S., & Titiunik, R. (2009). Housing, health, and happiness. *American Economic Journal: Economic Policy*, 1(1), 75–105. 10.1257/pol.1.1.75

[cit0006] Causes of urban poverty in India. Assessed on March 29 from https://www.habitatforhumanity.org.uk/blog/2018/08/causes-urban-poverty-india/

[cit0007] Corburn, J., Vlahov, D., Mberu, B., Riley, L., Caiaffa, W. T., Rashid, S. F., Ko, A., Patel, S., Jukur, S., Martínez-Herrera, E., Jayasinghe, S., Agarwal, S., Nguendo-Yongsi, B., Weru, J., Ouma, S., Edmundo, K., Oni, T., & Ayad, H. (2020). Slum health: Arresting COVID-19 and improving well-being in urban informal settlements. *Journal of Urban Health*, 97(3), 348–357. 10.1007/s11524-020-00438-6 32333243 PMC7182092

[cit0008] Das, M., Das, A., Giri, B., Sarkar, R., & Saha, S. (2021). Habitat vulnerability in slum areas of India–what we learnt from COVID-19? *International Journal of Disaster Risk Reduction*, 65, 102553. 10.1016/j.ijdrr.2021.102553 34513585 PMC8421084

[cit0009] De Haas, H. (2021). A theory of migration: The aspirations-capabilities framework. *Comparative Migration Studies*, 9(1), 1–35. 10.1186/s40878-020-00210-4 PMC7902564 33680858

[cit0010] Edition, F. (2011). Guidelines for drinking-water quality. *WHO Chronicle*, 38(4), 104–108. 6485306

[cit0011] Farmer, P. (2004). An anthropology of structural violence. *Current Anthropology*, 45(3), 305–325. 10.1086/382250

[cit0012] Fereday, J., & Muir-Cochrane, E. (2006). Demonstrating rigor using thematic analysis: A hybrid approach of inductive and deductive coding and theme development. *International Journal of Qualitative Methods*, 5(1), 80–92. 10.1177/160940690600500107

[cit0013] Government of India. (2011). Census of India 2011 & quot; provisional population totals. Accessed June 23, from http://censusindia.gov.in/2011-prov-results/data_files/india/paper_contentsetc.pdf.

[cit0014] Jayanthakumaran, K., Verma, R., Wan, G., & Wilson, E. (2019). Internal migration. In G. Wan (Ed.),*Urbanization and poverty in Asia: Dynamics and interrelationships* (p. 367). Springer Nature.

[cit0015] Mberu, B. U., Ciera, J. M., Elungata, P., & Ezeh, A. C. (2014). Patterns and determinants of poverty transitions among poor urban households in Nairobi, Kenya. *African Development Review*, 26(1), 172–185. 10.1111/1467-8268.12073

[cit0016] Mberu, B. U., Haregu, T. N., Kyobutungi, C., & Ezeh, A. C. (2016). Health and health-related indicators in slum, rural, and urban communities: A comparative analysis. *Global Health Action*, 9(1), 33163. 10.3402/gha.v9.33163 27924741 PMC5141369

[cit0017] Migration. Urbanization, and the family dimension. Assessed on February 2 from https://www.un.org/development/desa/family/wp-content/uploads/stes/23/2022/04/Migration-Urbanization-and-the-Family-Dimension-by-Bahira-Trask.pdf

[cit0018] Migration and its impact on cities. Assessed on March 20 from https://www3.weforum.org/docs/Migration_Impact_Cities_report_2017_low.pdf

[cit0019] National mission for sustainable agriculture. Assessed on March 3 from 4. https://nmsa.gov.in

[cit0020] National rural health mission assessed on March from https://nhm.gov.in/index1.php?lang=1&level=1&lid=49&sublinkid=969#:~:text=The%20National%20Rural%20Health%20Mission,population%2C%20especially%20the%20vulnerable%20groups

[cit0021] National urban health mission assessed on January from https://nhm.gov.in/index1.php?lang=1&level=1&sublinkid=970&lid=137

[cit0022] Ooi, G. L., & Phua, K. H. Urbanization and slum formation. *Journal of Urban Health*, 84(1), 27–34. 2007 May 1. 10.1007/s11524-007-9167-5 PMC1891640 17387618

[cit0023] Parida, J. K. (2019). Rural-urban migration, urbanization, and wage differentials in urban India. *Internal Migration, Urbanization and Poverty in Asia: Dynamics and Interrelationships*, 189–218. doi:10.1007/978-981-13-1537-4_8.

[cit0024] Park, Y. S., Konge, L., & Artino, A. R. (2020). The positivism paradigm of research. *Academic Medicine*, 95(5), 690–694. 10.1097/ACM.0000000000003093 31789841

[cit0025] Pawar, A. B., Mohan, P. V. T. K., & Bansal, R. K. (2008). Social determinants, suboptimal health behavior, and morbidity in urban slum population: An Indian perspective. *Journal of Urban Health*, 85(4), 607–618. 10.1007/s11524-008-9261-3 18404392 PMC2443249

[cit0026] A portrait of the chronically ill in America, (2001).Accessed on March 10 from. http://www.rwjf.org/f iles/publications/other/ChronicIllnessChartbook2001.pdf

[cit0027] Pradhan Mantri Fasal Bima Yojana. Assessed on January 26 from https://www.pmfby.gov.in

[cit0028] Pradhan Mantri Krishi Sinchayee Yojana (PMKSY). Assessed on January 3 from 3. https://pmksy.gov.in

[cit0029] Public distribution scheme. Assessed on March 1 from https://nfsa.gov.in/portal/PDS_page

[cit0030] PWC India. (2015). Forgotten voices: The world of urban children in India. Accessed from https://www.savethechildren.in/sci-in/files/79/79bfb888-7ed0-496e-b1e7-e71f7814ea7e.pdf

[cit0031] Rural. Assessed on February 5 from htps://www.india.gov.in/topics/rural

[cit0032] Shivayogi, P. (2013). Vulnerable population and methods for their safeguard. *Perspectives in Clinical Research*, 4(1), 53. 10.4103/2229-3485.106389 23533983 PMC3601707

[cit0033] United Nations. (2012). World urbanization prospects the 2011 revision. Accessed on June 23 from http://www.un.org/en/development/desa/population/publications/pdf/urbanization/WUP2 011_Report.pdf.

[cit0034] United Nations. (2018). & quot; 68% of the world population projected to live in urban areas by 2050, says UN & quot. Retrieved June 15, 2021, from https://www.un.org/development/desa/en/news/population/2018-revision-of-world-urbanization-prospects.html

[cit0035] Urbanisation, rural–urban migration and urban poverty. Assessed on January 17 from https://www.iied.org/sites/default/files/pdfs/migrate/10725IIED.pdf

[cit0036] Urban Secretariat, U.-H., & Branch, S. (2002). *Expert group meeting on urban indicators: Secure tenure, slums and global sample of cities*. UN-Habitat.

[cit0037] Usmani, G., & Ahmad, N. 2018. Health status in India: A study of urban slum and non-slum population. *Journal of Nursing Research and Practice*, 2(1), 9–14. January 18.

