# Supplementary material for: Exploring the complexities of slum vulnerability in Haryana, India: a qualitative research journey into economic, social, physical, and health dimensions
Source: Int J Qual Stud Health Well-being. 2024 Dec 15;20(1):2432692. doi: 10.1080/17482631.2024.2432692 (PMC11650438; doi:10.1080/17482631.2024.2432692)
Supplement: FGD Tool.pdf [file ZQHW_A_2432692_SM2799.pdf]

Annexure A:
Group Discussion Tool for Vulnerable Slums

I. General Details

Name of the State	
Name of the City	
Name and number of ward	
Identified vulnerable group	
Vulnerability Criteria(s) for Selected community/population	

INTRODUCTION AND INFORMED CONSENT

Namaste,

My name is ______________________________________ and I am working with (NAME OF ORGANIZATION/DEPARTMENT). Our organization/department is assisting the state government in conducting health vulnerability assessment as part of the National Urban Health Mission (NUHM) in various cities across the country.

One of the main objectives of the NUHM relates to ensuring that vulnerable population in a city have access to primary health care services. We are conducting this discussion to understand the prevalence of health and other related issues in the community. We would like to understand issues/challenges pertaining to access to the health services.

The information provided by you would be kept confidential and used only for the purpose of research and programme planning.

S. No.	Participant Name

Date of discussion:	
Name of facilitators:	
Name of recorder/s:	

VULNERABLE GROUPS AND COMMUNITY PROCESSES

II. About Vulnerable People as Community

S. No.	Questions and Probes
**1. General Information**	
1.1	Since when you all have been staying here?
1.2	Where do you all come from? Same place or distance places?
**2. Social Category**	
2.1	Do you identify yourself as being part of a specific community? If yes, please tell which community you belong to?<br>(*This question reveals the understanding of members about their community identity with respect to caste, religion, occupation, geography, or any other shared services*).
**3. Housing**	
3.1	What kind of houses are mostly available in your community (Pakka/Kutcha)?
	Do most of the people live in self owned or rented houses?
3.2	Are there any threats of being displaced from here? Of fire, or floods or physical dangers of any other sort.
**4. Occupation**	
4.1	What is the common occupation or occupations of your community? Are there any other occupational groups in your area? (list different types of occupation of the community).
4.2	Are people living in your employed throughout the year? If not, how many days on an average do they work for a year?

III. About Health Issues and Health Services

S. No.	Questions and Probes
**1. Health Issues**	
1.1	What are the general health problems that your community face?
1.2	What do you think, are the causes for your health problems?
**2. Access to Health Services**	
2.1	How far is the nearest health facility? Does the community face any problems in access to care in these facilities?
2.2	Where do people from the community generally go at the time of any health problems (Public or Private Health facility)? Give reasons for why you chose the specific health facility?
2.3	Kindly list the services provided in the nearest ***public*** facility?<br>(*Probe for availability of doctors, drugs, diagnostics, any other*)
2.4	Where do women often go for delivery services (Public/Private)? Why?<br>(*Probe reasons w.r.t to availability of services, access, quality of care, cost, etc.)*
2.5	Are there any outreach camps like Immunization, ANC camps being organised in your area? If yes, then what is the frequency for the same? Do women and children from your community go to avail services from these outreach camps? (*probe for reasons*)
2.6	Is there any frontline staff (ASHA/ANM etc.) assigned for your community?<br>(*Discuss to find in case there is an ASHA/ANM, is she from there community, how regularly she imparts the desired services and any other related issues*)
2.7	Are there patients suffering from Tuberculosis/Diabetes/Hypertension in your community? What is their experience with access to care and drugs?
2.8	Is there any insurance scheme available for the families in your community? Do you have a card for it? Which members of the family are covered and which are left out?<br>Do you know what the sum assured is and in which hospitals you are eligible to free care? Have you made use of the card to get any free services so far? If so the details? If you or family members have been ill but despite this unable to access free services- what are the reasons?

IV. Access to Other Services

S. No.	Questions and Probes
**1. Electricity Status**	
1.1	Do most of the houses have metered electricity connections?<br>If yes, how many hours' there is face power-cuts generally in a day?

S. No.	Questions and Probes
**2. Availability of Water**	
2.1	If there is availability of drinking water in your community? What are the different sources of water? Where do you go to if there are problems in access and how responsive are the authorities on this?
**3. Availability of Toilets**	
3.1	Are there toilets in your community (public or individual)? If yes, are they being used by the community? If no, why?
**4. Waste Management System**	
4.1	Where do people dispose their solid wastes/rubbish in your locality? If authority regular clears the waste from your locality? (*probe to understand if there is a designated place for waste disposal, frequency of clearance of waste and any other issue associated with waste)*
**5. Public Distribution System**	
5.1	Do people have ration card in your community? If yes, do they utilize the service of Fair price Shop? (*if, yes, probe for quality and availability of service, if no, probe for reasons for not utilizing the services)*
**6. Anganwadi Centre**	
6.1	Do you have an ICDS Centre (Anganwadi) in the neighbourhood? If yes, what service are available at the AWC? Do mothers and children regularly avail services from them? If no, why?
**7. Schools**	
7.1	Do children go to school in your community? If no, why? (*Probe for non-availability of school, issue pertaining to access, quality, drop-out, child labourer, etc.)*<br><br>If yes, where does most of the children go for schooling (private/public/NGO/Trust school)? Why? How far is the school?
7.2	Are there school health programmes implemented in your community? If yes, how often does doctor or nurse come- are they aware of the school health programme at all?

V. NGOs in Action

S. No.	Questions and Probes
**1.**	Are there NGOs/CBOs in your areas?
**2.**	What are the different interventions NGOs/CBOs are working in your areas?
**3.**	Are there community groups (SHGs/others) made by NGOs/CBOs in your area? Please describe since how long they exist, what different activities it does and how?
